# Grandiose and vulnerable narcissism, identity integration and self-control related to criminal behavior

**DOI:** 10.1186/s40359-021-00697-1

**Published:** 2021-12-03

**Authors:** S. Bogaerts, C. Garofalo, E. De Caluwé, M. Janković

**Affiliations:** 1 Department of Developmental Psychology, Tilburg University, P.O. Box 90153, 5000 LE Tilburg, the Netherlands; 2 Fivoor Science and Treatment Innovation (FARID), Rotterdam, the Netherlands

**Keywords:** Vulnerable narcissism, Grandiose narcissism, Criminal behavior, Identity integration, Forensic outpatients, Self-control

## Abstract

**Background:**

Although systematic research on narcissism has been conducted for over 100 years, researchers have only recently started to distinguish between grandiose and vulnerable narcissism in relation to criminal behavior. In addition, there is some evidence suggesting that identity integration and self-control may underlie this association. Therefore, the present study aimed to develop a theory-driven hypothetical model that investigates the complex associations between grandiose and vulnerable narcissism, identity integration, self-control, and criminal behavior using structural equation modeling (SEM).

**Methods:**

The total sample (*N* = 222) included 65 (29.3%) individuals convicted of criminal behavior and 157 (70.7%) participants from the community, with a mean age of 37.71 years (*SD* = 13.25). Criminal behavior was a grouping variable used as a categorical outcome, whereas self-report questionnaires were used to assess grandiose and vulnerable narcissism, self-control, and identity integration.

**Results:**

The overall SEM model yielded good fit indices. Grandiose narcissism negatively predicted criminal behavior above and beyond the influence of identity integration and self-control. In contrast, vulnerable narcissism did not have a direct significant effect on criminal behavior, but it was indirectly and positively associated with criminal behavior via identity integration and self-control. Moreover, grandiose narcissism was positively, whereas vulnerable narcissism was negatively associated with identity integration. However, identity integration did not have a direct significant effect on criminal behavior, but it was indirectly and negatively associated with criminal behavior via self-control. Finally, self-control was, in turn, negatively related to criminal behavior.

**Conclusions:**

We propose that both subtypes of narcissism should be carefully considered in clinical assessment and current intervention practices.

**Supplementary Information:**

The online version contains supplementary material available at 10.1186/s40359-021-00697-1.

## Introduction

The antecedents of criminality have long been of interest to criminological researchers, as well as factors that mediate the links between them (e.g., [1]). However, most of the existing studies on personality characteristics and abilities that contribute to the development of criminal behavior have focused on single factors in relation to offending, and integration among studies has often occurred post-hoc via logical inferences (e.g., because construct X is related to construct Y, which in turn is related to criminal behavior, an indirect effect can be logically expected). In the current study, we hence proposed and tested a theory*-*driven model that focuses on the interplay between narcissism, identity integration, and self-control, in the explanation of criminal behavior*.*

Despite numerous studies on narcissism, no consensus has been reached on a widely accepted definition of narcissism [2–4]. However, over the past 20 years, there has been broad recognition of the need to differentiate between different types of narcissism that can be roughly divided into narcissistic grandiosity and narcissistic vulnerability [4–6]. Although both subtypes of narcissism share a common deeper foundation, such as self-centeredness [7], they can have very different manifestations. Grandiose narcissism as a pathological characteristic manifests itself in exaggerated self-esteem, grandiosity and an unrealistic sense of superiority, as well as admiration seeking, entitlement and arrogance [4, 6, 8]. Most experts agree that grandiose narcissism is more a characteristic of the Narcissistic Personality Disorder, as defined by the *Diagnostic and Statistical Manual of Mental Disorders*, 5th Edition, Section II (DSM-5; [9]), than vulnerable narcissism is [3, 5, 10]. In contrast, vulnerable narcissism entails pronounced self-absorbedness, low self‐esteem, hypervigilance, shyness, social withdrawal and emotional hypersensitivity [11, 12]. Recent studies have shown that grandiose narcissism is less harmful to mental health, while vulnerable narcissism is associated with psychological problems and the use of rather inappropriate emotion regulation strategies, such as aggression and repression [13].

In general, research suggests that narcissism is quite overrepresented in samples of violent offenders (e.g., [14–17]), and positively associated with criminal behavior [18, 19]. However, in the field of forensic psychology, researchers have only recently begun to investigate the difference between grandiose and vulnerable narcissism and no research to date has investigated how both forms differ from one another concerning criminal behavior. Yet, some indirect evidence emerges from studies on grandiose and vulnerable narcissism in relation to aggression, more specifically proactive and reactive aggression. While the results have been somewhat mixed*,* the available evidence suggests that narcissistic grandiosity is associated with both forms of aggression, while narcissistic vulnerability is associated only with reactive aggression (e.g., [20–22]). Compared to vulnerable narcissism, grandiose narcissism has also been more strongly associated with a wide variety of impulsivity-related externalizing behaviors, such as gambling [23], substance use [24], antisocial behaviors [25], and proactive aggression [26]. Individuals with higher levels of grandiose narcissism may have excessive confidence in their competencies and take more risks [27], probably due to their excessively active reward-oriented system (e.g., [28]). They focus more on positive outcomes and do not estimate chances and outcomes in a realistic way [23]. Additionally, aggression in individuals with higher levels of grandiose narcissism is usually seen as a self-enhancing strategy with the aim of restoring or enforcing a sense of superiority [29, 30]. However, there is also contrasting evidence suggesting that individuals with high narcissistic vulnerability are more likely to display aggressive behavior than individuals high on grandiosity (e.g., [31, 32]). For example, Krizan and Johar [31] found that narcissistic vulnerability (but not grandiosity) has particularly shown to be a powerful driver of rage, hostility, and aggressive behavior, fueled by suspiciousness, dejection, and angry rumination. The fragmented sense of the self and desperate need for external appreciation predisposes individuals with higher levels of vulnerable narcissism to experience shame about their narcissistic needs and unrestrained anger towards those who exposed their weaknesses [33]. This, in turn, triggers “narcissistic rage” that can further promote aggressive behavior [31]. Due to inconsistency and a scarcity of empirical evidence*,* additional research is needed to uncover whether and how these two subtypes of narcissism are associated with criminal behavior. Indeed, previous research has mainly focused on the link between narcissism and aggressive behavior in samples of the general population. Possible relations of different variants of narcissism with more severe forms of violent behavior (e.g., sanctioned by society) remained largely understudied.

Likewise, little is known about the mechanisms underlying the association between narcissism and criminality. According to Stern [34], the narcissistic individual is often attuned to what other individuals feel and think. This notion is closely related to the core aspect of identity, namely the fact that the individual is partly determined by interaction with his environment and must develop the ability to act effectively as an independent subject in that environment.

Identity refers to how a person defines the self and understands intimate relationships and social interactions with the social world. Identity formation is a process of alternating phases of ‘crisis and commitment’ that occur especially during adolescence [35]. Identity integration can be defined as a coherence of identity; the capacity to see oneself and one's life as stable, integrated and purposive [36]. In contrast, identity diffusion is characterized by a lack of normative commitment and reflects difficulties in maintaining a relatively constant set of goals [37]. Notably, identity diffusion does not occur in a vacuum; rather, it is an important feature that is associated with various personality dysfunctions and characterizes personality pathology [38–40]. According to the DSM-5 Section III [9], significant impairments in self-identity (e.g., unstable self-image, inconsistencies in values, goals, and appearance) and interpersonal functioning (e.g., being insensitive to others, inconsistent, detached, or abusive style of relating) are the main characteristics of personality disorders. In particular, identity diffusion (i.e., incoherent self-image, self-fragmentation) is one of the core components of a narcissistic personality disorder [41, 42]. Narcissistic individuals show excessive dependency on others for identity; they need constant external support and attention to maintain their self-esteem, and self-esteem problems often shift between inflated and deflated self-appraisal [43].

Despite theoretical elaboration of the role of identity in narcissism, there is little empirical research on the association between narcissism and identity integration. However, available evidence suggests that narcissistic traits [41, 44], and in particular narcissistic vulnerability [39, 40, 45], are associated with higher identity instability (i.e., a weak sense of the self). For example, Dashineau et al. [39] found that narcissistic vulnerability was associated with all forms of dysfunction (e.g., well-being, self-control, and everyday life tasks), while grandiosity was associated with specific deficits in interpersonal functioning. However, after accounting for shared variance in vulnerability, grandiosity was not associated with most aspects of poor functioning and was positively associated with better functioning in some areas, such as life satisfaction. Similarly, Huxley et al. [40] found that vulnerable narcissism was associated with impairment in self- and relational functioning, while grandiosity predicted higher self-functioning. More research is needed to investigate how both grandiose and vulnerable narcissism are associated with identity integration.

Furthermore, it has been shown that identity diffusion can result in feelings of emptiness, deviant behavior and superficiality, or other maladaptive outcomes, such as poor impulse control [41, 42]. In the identity-value model, Berkman et al. [46] proposed that identity plays a crucial role in self-control. By its definition, identity is a relatively stable mental representation of personal and intrapersonal values, priorities, and roles. Therefore, individuals are more prone to associate their identity with long-term goals than with short-term impulses. According to this model, self-control is defined as a decision-making process that compares the subjective value of two options and selects the option with the highest value [46]. Therefore, individuals with more integrated identity are better at making choices that are relevant to their long-term goals over short-term impulses, meaning they are better at self-control.

Self-control is conceptualized as the capacity to tolerate, use and control one’s own emotions and impulses [36]. Research has shown that the degree of self-control is positively associated with adaptive correlates in various life domains, such as academic and professional success, healthier and more sustainable intimate relationships, closer social networks, greater self-awareness, empathy, and more proactive health behaviors (e.g., regular medical check-ups; [47]). In contrast, a lack of self-control is linked to a wide range of antisocial and deviant behaviors [48–51], and a variety of negative life outcomes, such as criminal victimization, poor health, and financial difficulties (e.g., [52–54]).

According to the general theory of crime [55], a lack of self-control is the main factor behind all criminal acts [56, 57], although in this theory self-control was conceptualized in broader terms as “the differential tendency of people to avoid criminal acts whatever the circumstances in which they find themselves” [55 p87]. A lack of self-control was thus characterized by impulsive behavior towards others, physical risk-taking and shortsightedness, and can give rise to criminal acts in interaction with situational opportunities [55].

In sum, there is evidence that grandiose and vulnerable narcissism contribute to disintegrated identity and criminal behavior. In addition, there are indications that identity diffusion is directly associated with criminal behavior and that this association is mediated by self-control. Several studies have reported bivariate associations between pairs of these constructs, as previously reviewed. However, to our knowledge, no studies so far have investigated whether identity integration and self-control sequentially mediate the association between grandiose and vulnerable narcissism and criminal behavior.

## The present study

**Fig. 1:**
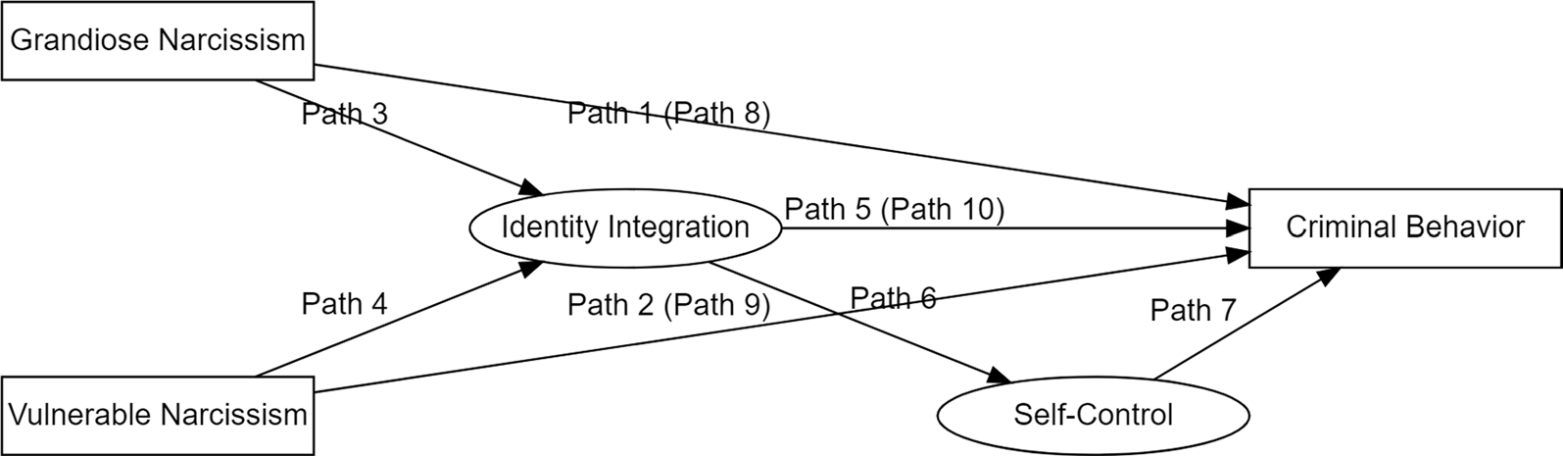
Hypothetical conceptual model. Indirect paths are in the brackets

Therefore, the goal of the present study was to develop a theory-driven hypothetical model by using structural equation modeling (SEM) in a cross-section design. Although we cannot test causal relationships in a cross-sectional design, SEM is widely used in social science research to test a hypothetical conceptual model [58–60]. In this model (see Fig. 1), complex associations between grandiose and vulnerable narcissism, identity integration, self-control, and criminal behavior were investigated with specific theory-driven hypotheses about the sequential mediation of identity integration and self-control in the link between narcissism and criminal behavior. First, based on the available evidence [e.g., 20–22, 25, 26], we hypothesized that both grandiose (path 1) and vulnerable narcissism (path 2) would be directly and positively associated with criminal behavior. However, due to the mixed empirical findings of the extent to which grandiose and vulnerable narcissism contribute to violent offending, we had no specific hypotheses as to which of both narcissistic subtypes on criminal behavior would be stronger. Second, since identity diffusion is one of the key features of a narcissistic personality disorder [41, 42], we hypothesized that both grandiose and vulnerable narcissism would be directly and negatively associated with identity integration (path 3 and path 4, respectively). Nonetheless, this link might be expected to be weaker for grandiose narcissism, as grandiose narcissism has been documented to be associated with a narrower range of poor identity functioning and better life satisfaction compared to vulnerable narcissism [e.g., 39, 40]. Furthermore, it has been shown that identity diffusion can lead to deviant behavior and a range of maladaptive outcomes such as poor impulse control [48–51]. It also plays a vital role in self-control and individuals with a more integrated identity are better at self-control [46]. Therefore, it was hypothesized that identity integration would have a direct negative effect on criminal behavior (path 5) and a direct positive effect on self-control (path 6). Lastly, previous research has shown that a lack of self-control is associated with a wide range of antisocial and deviant behavior [48–51] and is the main factor behind all criminal acts [55]. Hence, it was hypothesized that self-control would have a direct negative effect on criminal behavior (path 7). Despite supporting these direct links, the review literature also indicates that there may be indirect effects between these variables. Therefore, a series of indirect effects was assumed. Identity integration and self-control were hypothesized to mediate the association between grandiose narcissism and criminal behavior (path 8 [i.e., paths 3, 6, 7]) and between vulnerable narcissism and criminal behavior (path 9 [i.e., paths 4, 6, 7]). Finally, it was hypothesized that self-control would mediate the association between identity integration and criminal behavior (path 10 [i.e., paths 6 and 7]).

## Methods

### Procedure

Master level psychology students who did their clinical internship in three outpatient forensic psychiatric centers recruited the individuals convicted of criminal behavior. All offenders undergo mandatory outpatient treatment in these forensic psychiatric centers which was imposed by the judge as a result of a committed offense. Treatments mainly focused on aggression and emotion regulation based on cognitive behavioral therapy. During a therapeutic session, potential participants were asked if they were willing to participate in the study and also received an information letter. In that letter, it was clearly stated that participation was voluntary and that refusing to participate would not influence the participant’s treatment in any way. The participants had approximately one week to consider their potential participation. Participants who agreed to participate in this study were asked to complete a set of psychosocial questionnaires and were rewarded with monetary compensation of five euros. The questionnaires were completed during a treatment session to cause as little burden as possible to the offenders.

Furthermore, 22 Dutch bachelor and master level psychology students collected data in the community from October 2014 to March 2015. The survey was administered via the Qualtrics platform and made available to the general population through publishing on social media. Participants had to be at least 18 years old and have sufficient knowledge of the Dutch language. Control subjects were matched with the delinquent population on two characteristics, namely age and level of education. Participants with a university degree were excluded from the control group because this category did not appear in a sample of delinquents. After being informed of the goal and procedure of the study by an information letter, all participants signed informed consent and participated voluntarily in the study without receiving financial compensation. Before completing the survey, which included a set of validated psychosocial questionnaires, the participants were asked whether they had ever been convicted of an offense and whether a psychologist, psychotherapist or other care provider had treated them in the past 3 years. If the answer was 'yes', they could not participate in the study. After this, the questionnaires could be completed.

To guaranty anonymity, all participants were instructed to return the questionnaires in a sealed envelope after completion. The sealed envelopes and consent statements were given to the student's supervisor. The informed consent was removed before the data encoding. All procedures involving human participants were performed in accordance with the ethical standards of the institutional and/or national research committee and with the 1964 Helsinki Declaration and its later amendments or comparable ethical standards. The Committee of Scientific Research of FPC Kijvelanden and the local university ethics review board approved the study.

### Participants

The total sample included 222 male participants. Of this sample, 65 (29.3%) were individuals convicted of criminal behavior and 157 (70.7%) were controls from the community. The mean age of the participants was 37.71 years (*SD* = 13.25), ranging from 20 to 60. Most of the participants (67.6%, *n* = 150) had a Dutch nationality, lived alone (28.2%, *n* = 62) and had an income from paid employment (65.4%, *n* = 138). The most common finished level of education was intermediate vocational education/MBO (31.1%, *n* = 69), next to higher professional education/HBO (28.4%, *n* = 63), higher general secondary education/HVO (9%, *n* = 20) and secondary education/VWO (9%, *n* = 20). The index offenses of individuals convicted of criminal behavior included a variety of violent offenses: physical aggression (45.3%, *n* = 29); domestic violence (31.3%, *n* = 20); verbal aggression (20.3%, *n* = 13); and other offenses (3.1%, *n* = 10). More details about the demographic characteristics of the two groups can be found in the appendix (see Additional file 1: Table S1). The questionnaire characteristics (including *F* tests) of the two groups are shown in Table 1. Compared to the control group, the group of individuals convicted of criminal behavior showed significantly higher levels of vulnerable narcissism and lower levels of grandiose narcissism, self-control and identity integration.

**Table 1:** Questionnaire characteristics of the two groups

	Mean (*SD*)		Test statistics	*p*
	Offenders	Controls		
Grandiose narcissism	53.89 (10.33)	60.30 (7.35)	*F*(1,215) = 26.855	< .001
Vulnerable narcissism	45.22 (10.61)	38.91 (10.89)	*F*(1,218) = 15.588	< .001
Self-control	28.95. (7.94)	42.43 (5.51)	*F*(1,220) = 209.462	< .001
Identity integration	34.67 (8.99)	43.26 (5.85)	*F*(1,220) = 71.093	< .001

*SD* = standard deviation

### Measures

#### The Dutch Narcissism scale

Narcissism was measured with the Nederlandse Narcisme Schaal ([Dutch Narcissism Scale]; NNS; [61]). The NNS is based on the Narcissistic Personality Inventory [62, 63] and on the Hypersensitive Narcissism scale [64]. The NNS is a Dutch questionnaire that consists of 35 items measuring three different types of narcissism: vulnerable (11 items), grandiose (12 items) and isolation (12 items). The isolation subscale was not used in this study given its misalignment with our theoretical model. All items are rated on a seven-point Likert scale ranging from 1 “that is certainly not the case” to 7 “that is certainly the case”, with higher scores indicating greater levels of narcissism. An example of a vulnerable narcissism item is: “Small remarks of others can sometimes easily hurt my feelings”. An example of a grandiose narcissism item is: “Sometimes I feel like I got lucky with who I am anyway” [61]. The validity of the Dutch NNS was supported by its relations with age, self-esteem, burnout, and empathy [61], meaning of life [65, 66], and depression [66], which paralleled findings obtained with other narcissism inventories. Past research has also demonstrated good internal consistency (Cronbach’s α) of both scales, ranging from 0.71 to 0.77 for grandiose narcissism and from 0.77 to 0.87 for vulnerable narcissism. In the current sample, the internal consistency (Cronbach’s alpha) of the grandiose and vulnerable narcissism scales was acceptable to good with Cronbach’s alpha of α = 0.71 and α = 0.81, respectively. For more details about item means and factor loadings, see Additional file 1: Table S2 in the appendix.

#### The severity indices of personality problems: short form

The Severity Indices of Personality Problems—Short Form (SIPP-SF; [36]) is a 60-item self-report questionnaire derived from the SIPP-118. The SIPP-SF measures five domains of maladaptive personality functioning, namely: self-control (12 items), identity integration (12 items), relational capacities (12 items), responsibility (12 items) and social concordance (12 items). All items are rated on a four-point Likert scale ranging from 1 “fully disagree” to 4 “fully agree”, with higher scores corresponding with greater levels of functioning. For the purpose of the present study, only the domains self-control and identity integration of the SIPP-SF were used. The former assesses the capacity to tolerate, use and control one’s own emotions and impulses, whereas the latter assesses the capacity to see oneself and one’s own life as stable, integrated and purposive [36]. In the current sample, both domains (i.e., self-control and identity integration) showed excellent internal consistency, with a Cronbach’s α = 0.93 and α = 0.92, respectively. For more details about item means and factor loadings, see Additional file 1: Table S3 in the appendix.

#### Criminal behavior

Criminal behavior was used as a grouping variable (0 = community participants, 1 = sample of offenders), and defined as being convicted of one or more of the following offenses: physical aggression, domestic violence, verbal aggression, violent property offense and stalking. Because we could not have any a-priori theoretical expectation about distinct links with each type of offense as we had no information about the criminal history, and also to maintain statistical power, we deliberately chose this grouping variable of overall offending (referring to violent criminal behavior).

### Statistical analysis

All analyses were computed by using the lavaan package in R [67, 68] and SPSS version 25.0 [69]. To determine the bivariate associations between continuous indicators and criminal behavior (i.e., binary outcome variable), we computed the point-biserial correlations. Furthermore, to investigate the interrelation of grandiose and vulnerable narcissism, identity integration, self-control and criminal behavior, path analysis was applied. Path analysis is a subset of SEM and only deals with observed variables. Path analysis was used to investigate whether the assumed theoretical model corresponds to the cross-sectional empirical model that has been studied. A model was estimated with Maximum Likelihood Estimation, which searches for parameter estimates that make probability for observed data maximal [70]. The model fit was evaluated using the following fit indices: Comparative Fit Index (CFI) and Standardized Root Mean Square Residual (SRMR). The CFI compares the fit of a target model to the fit of a baseline model. Values exceeding 0.90 indicate a well-fitting model. The SRMR represents the square-root of the difference between the residuals of the sample covariance matrix and the hypothesized model. A value less than 0.08 suggests a good model fit [71]. The minimum sample size for conducting SEM is at least five observations per estimated parameter [72], which means that we had enough statistical power to detect statistically significant effects. Lastly, missing values were handled with pairwise deletion.

## Results

Correlations between all study variables including age are shown in Table 2. Grandiose narcissism was negatively associated with criminal behavior and positively associated with identity integration and self-control. On the contrary, vulnerable narcissism was positively associated with criminal behavior and negatively associated with identity integration and self-control. Moreover, identity integration was negatively associated with criminal behavior and positively associated with self-control. Finally, self-control was negatively associated with criminal behavior. Considering age, it was negatively associated with both forms of narcissism and positively associated with self-control.

**Table 2:** Point-Biserial correlations between the different variables

Variable	1	2	3	4	5	6
1. Age	1					
2. Grandiose narcissism	− .161*	1				
3. Vulnerable narcissism	− .311**	− .018	1			
4. Self-control	.158*	.336**	− .416**	1		
5. Identity integration	.111	.436**	− .467**	.642**	1	
6. Criminal behavior	− .036	− .333**	.258**	− .698**	− .494**	1

*Correlation is significant at the .05 level (2-tailed). ** Correlation is significant at the .01 level (2-tailed)

**Fig. 2:**
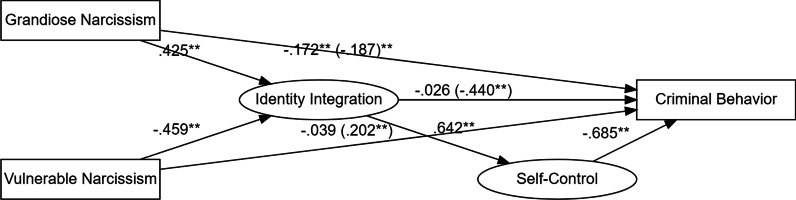
Standardized model results. ** Association is significant at the .01 level (2-tailed). Indirect effects are in the brackets

Subsequently, path analysis was performed to investigate the direct and indirect associations between narcissism (i.e., grandiose and vulnerable), identity integration, self-control, and criminal behavior. The data fit sufficiently well with the hypothetical conceptual model based on CFI = 0.98 and SRMR = 0.04. Results are summarized in Table 3 and Fig. 2. Grandiose narcissism had a significant direct negative path to criminal behavior (path 1), whereas vulnerable narcissism appeared to be non-significantly associated with criminal behavior (path 2). Furthermore, grandiose narcissism had a significant direct positive path to identity integration (path 3), whereas vulnerable narcissism had a significant direct negative path to identity integration (path 4). In addition, identity integration did not have a significant direct path to criminal behavior (path 5), but it had a significant direct positive path to self-control (path 6). Self-control, in turn, had a significant direct negative path to criminal behavior (path 7). Moreover, considering mediating effects, the results showed that identity integration and self-control partially mediated a negative association between grandiose narcissism and criminal behavior (path 8) and fully mediated a positive association between vulnerable narcissism and criminal behavior (path 9). Finally, identity integration was indirectly and negatively associated with criminal behavior through self-control (path 10).

**Table 3:** Unstandardized and standardized model results

	Estimate	S.E	Std. all	*p*
*Criminal behavior*				
Grandiose narcissism (path 1)	− 0.009	0.002	− 0.172	< .001
Vulnerable narcissism (path 2)	0.002	0.002	0.039	.418
Identity integration (path 5)	− 0.002	0.004	− 0.026	.682
Self-control (path 7)	− 0.035	0.003	− 0.685	< .001
*Identity integration*				
Grandiose narcissism (path 3)	0.382	0.047	0.425	< .001
Vulnerable narcissism (path 4)	− 0.326	0.037	-0.459	< .001
*Self-control*				
Identity integration (path 6)	0.712	0.057	0.642	< .001
Indirect effect 1 (path 8)	− 0.010	0.002	− 0.187	< .001
Indirect effect 2 (path 9)	0.008	0.001	0.202	< .001
Indirect effect 3 (path 10)	− 0.025	0.003	− 0.440	< .001

S.E. = standard error; Std. all = all variables are standardized

## Discussion

The present study was the first to investigate the complex associations between grandiose and vulnerable narcissism, identity integration, self-control, and criminal behavior by using SEM. We hypothesized that both grandiose and vulnerable narcissism would have a direct positive effect on criminal behavior and a direct negative effect on identity integration. In addition, we expected identity integration to have a negative effect on criminal behavior, and a positive effect on self-control. Lastly, self-control was expected to be negatively associated with criminal behavior. Furthermore, different mediating effects between these associations were also hypothesized, in a sequential model connecting narcissism to identity integration, self-control, and criminal behavior, in this order. In addition, we also expected that self-control would mediate the association between identity integration and criminal behavior. Overall, the path analysis showed that the empirical model fits well with the proposed theoretical model. However, on the path-level, the results indicated that our expectations were not entirely supported.

Specifically, contrary to our expectations, we found that grandiose narcissism was not positively, but directly negatively associated with criminal behavior (path 1), whereas vulnerable narcissism did not have a significant direct effect on criminal behavior (path 2), despite a small but significant bivariate association. However, by inspecting the indirect effects, it should be noted that vulnerable narcissism was significantly positively associated with criminal behavior, but only via identity integration and self-control (path 9). The same indirect effect was significant for grandiose narcissism as well, yet in the opposite direction, however, without diminishing the direct effect of grandiose narcissism on criminal behavior (path 8). This could lead to the conclusion that higher levels of identity integration and self-control partially explained a negative association between grandiose narcissism and criminal behavior, and lower levels of identity integration and self-control fully explained the positive association between vulnerable narcissism and criminal behavior. Our result is in line with previous finding showing that grandiose narcissism is not necessarily associated with criminal behavior [13] and that narcissistic vulnerability, but not grandiosity, is a stronger indicator of aggressive behavior and hostility ([31, 32, 73]; but see [20, 22]). People high on narcissistic vulnerability often use inappropriate emotion-regulating strategies, which might lead to more anger and aggression (e.g., 13, [31, 32]). However, in the present study vulnerable narcissism predicted criminal behavior only indirectly.

A lack of a direct effect of vulnerable narcissism on delinquency (path 2) in our study might be explained by the design of the study. In other words, it is likely that previous studies that found a direct association between vulnerable narcissism and criminal behavior did not include mediators in the model. Here, with identity integration and self-control in our model, all association between vulnerable narcissism and criminal behavior goes through identity integration and self-control. It is not necessary that these individuals do not express aggression openly/directly, as the criminal behavior itself can be overt and direct. The statistical effect is not direct because we control for identity and self-control, which may be mechanisms linking vulnerable narcissism and criminal behavior.

Furthermore, the present study revealed that higher levels of grandiose narcissism and lower levels of vulnerable narcissism were associated with a more integrated identity (path 3 and 4, respectively). Consistent with our hypothesis, we found that vulnerable narcissism was associated with lower levels of identity integration. It might be that individuals high on narcissistic vulnerability have a lower integrated identity because they are more likely to maintain their self-esteem and to modulate their fragile ego by relying on the social approval of significant others [74]. It has also been shown that, with positive feedback, individuals with high levels of vulnerable narcissism can hide the negative and shameful self-image, but when external feedback is perceived as negative, they are forced to face their negative self-image and are deeply ashamed. In contrast, negative feedback does not affect the positive self-image of individuals with grandiose narcissistic traits [75].

Contrary to our expectations, grandiose narcissism was positively associated with identity integration (path 3) namely with a self-representation of oneself and one’s own life as stable, integrated, and purposive. It might be that individuals high on narcissistic grandiosity have a higher integrated identity because they are more likely to maintain their self-esteem by employing overt strategies, such as self-enhancement and devaluation of others [30]. The result may fit into previous studies showing that individuals with grandiose narcissistic traits are better adjusted compared to individuals with vulnerable narcissistic traits [39, 40, 45, 76–78]. For example, Ng et al. [76] found that grandiose narcissism predicted higher life satisfaction and lower perceived stress, whereas vulnerable narcissism showed the opposite pattern. It has been also shown that the agentic extraversion, a characteristic of grandiose narcissism (i.e., a tendency toward assertiveness, persistence, and achievement), may serve as a protective factor against psychopathology and thus contribute to higher well-being and the “happy face” of narcissism [77]. This could explain why individuals high on grandiosity are satisfied with their lives, although they remain potentially harmful to others [45].

Furthermore, identity integration did not have a direct significant negative effect on criminal behavior (path 5), which is not in line with our hypothesis. However, identity integration was significantly negatively associated with criminal behavior, but only indirectly via self-control (path 10). Due to the fact that there was a significant negative correlation between identity integration and criminal behavior, this could lead to the conclusion that self-control fully explained the association between identity integration and delinquency. Indeed, it has been shown that individuals with higher identity integration have better self-control and are therefore less likely to engage in criminal behavior [46].

In support of this evidence, we found that better-integrated identity was associated with higher levels of self-control (path 6), which in turn was negatively related to delinquency (path 7). Identity can be seen as a strong and enduring source of value, which has an important role in determining self-regulation and self-control outcomes [46]. Our result corresponds with previous findings showing that lower identity integration can be manifested through poor self-control [41, 46]. In addition, a lack of self-control has been linked to a wide range of antisocial and deviant behaviors [46–51, 56]. Finally, our findings also give support to the general theory of crime [55], in which a lack of self-control represents the most important explanatory factor behind criminal behaviors.

## Limitations

Several limitations should be considered while interpreting the results of the present study. First, the current study was limited by operationalizing criminal behavior as a dichotomous variable, as well as by a relatively small sample of 65 offenders and 157 controls, which might negatively affect statistical power and effect size. Second, the study sample included only male participants and therefore our findings are not generalizable to the population of females. In addition, convenience sampling was used to recruit the subsample of controls and hence the generalizability of the findings cannot be entirely justified. Third, to maintain statistical power, we did not include any covariates in the analysis, which may also influence the results. In the current sample, however, age was significantly associated with both forms of narcissism and with self-control. There were also significant differences in social status, educational level and income between controls and offenders (Additional file 1: Table S1). Future studies may consider including age and other demographic characteristics as covariates when examining these complex associations. Furthermore, different narcissism inventories operationalize grandiose narcissism differently; therefore, we can only conclude that the effects reported relate to the NNS operationalization of narcissism and call for replications with other measures of this construct. Lastly, the design of the study was cross-sectional, which does not allow us to draw causal inferences about the complex associations between grandiose and vulnerable narcissism, identity integration, self-control, and criminal behavior.

## Research and clinical implications

Despite the limitations mentioned above, this study could have important research and clinical implications. To the best of our knowledge, the interrelation of identity integration, narcissism, and self-control explaining criminal behavior has never been tested before. In this study, we emphasized the role of personality pathology in the development of disintegrated identity. In addition, this study demonstrated the importance of considering identity integration and self-control as significant factors underlying the association between narcissism and criminal behavior. Future studies should investigate the long-term relations between grandiose and vulnerable narcissism, identity integration, self-control, and criminal behavior. The findings of the current study may be of significant value for future intervention practices. So far, most studies on narcissism in forensic psychiatry have treated narcissism as a unidimensional construct. However, there is some evidence that grandiose and vulnerable narcissism should be treated independently, as they are differently associated with adverse outcomes, such as criminal behavior, as well as victimization [79]. Therefore, the differences between these two subtypes of narcissism should be carefully considered in clinical assessment and intervention practices.

## Conclusion

This study can deepen our understanding of the complex associations between different aspects of narcissism, identity integration, self-control, and criminal behavior. In particular, the findings of the present study revealed that grandiose narcissism can be seen as a better-adjusted subtype of narcissism, as it was associated with higher identity integration and non-criminal behavior. In contrast, vulnerable narcissism was associated with low identity integration and indirectly associated with criminal behavior. Moreover, our study showed that identity integration and self-control are important mediators in the association of both grandiose and vulnerable narcissism with criminal behavior. Finally, this research confirmed the importance of identity integration in potentially contributing to self-control, which in turn is highly relevant for deterring criminal behavior. Researchers may wish to confirm our conclusions in a larger and more representative sample, and the current study serves as a good starting point for further work.

## Supplementary Information

**Additional file 1**. Supplementary tables.

## Data Availability

The datasets used and/or analyzed during the current study are available from the corresponding author on reasonable request.

## Abbreviations

SEM: Structural equation modeling
NNS: Nederlandse Narcisme Schaal [Dutch Narcissism Scale]
SIPP-SF: Severity indices of personality problems – short form
SIPP-118: Severity indices of personality problems – 118
CFI: Comparative fit index
SRMR: Standardized root mean square residual
