# Supplementary material for: Grandiose and vulnerable narcissism, identity integration and self-control related to criminal behavior
Source: BMC Psychol. 2021 Dec 3;9:191. doi: 10.1186/s40359-021-00697-1 (PMC8641202; doi:10.1186/s40359-021-00697-1)
Supplement: Supplementary file 1 — Additional file 1. Supplementary tables. [file 40359_2021_697_MOESM1_ESM.docx]

**Supplementary Materials**

**Table S1**

*Demographic Characteristics*

|  | Offenders | Controls | Test Statistic |
| --- | --- | --- | --- |
| Mean age at the time of survey (*SD*) | 36.97 (9.85) | 38.02 (14.43) | *F*(1,219)=.594 |
| Social status (*N* (%)) |  |  | *Χ^2^*(8)=40.939^***^ |
| Living with relatives | 7 (10.9%) | 25 (16.0%) |  |
| Living with relatives with children | 1 (1.6%) | 2 (1.3%) |  |
| Single | 32 (50.0%) | 30 (19.2%) |  |
| Single with children | 8 (12.5%) | 4 (2.6%) |  |
| Married/living together | 5 (7.8%) | 35 (22.4%) |  |
| Married/living together with children | 10 (15.6%) | 48 (30.8%) |  |
| Retirement home or other institutes | 1 (1.6%) | 0 (0%) |  |
| Newly assembled family with or without children | 0 (0%) | 4 (2.6%) |  |
| Other | 0 (0%) | 8 (5.1%) |  |
| Level of education (*N* (%)) |  |  | *Χ^2^*(10)=78.548^***^ |
| None | 5 (7.7%) | 1 (0.6%) |  |
| Primary education | 11 (16.9%) | 1 (0.6%) |  |
| Lower secondary education | 2 (3.1%) | 1 (0.6%) |  |
| Lower vocational education/LBO | 10 (15.4%) | 5 (3.2%) |  |
| Corporate social responsibility/MVO | 10 (15.4%) | 4 (2.5%) |  |
| School of higher general secondary education/HVO | 1 (1.5%) | 19 (12.1%) |  |
| Intermediate vocational education/MBO | 19 (29.2%) | 50 (31.8%) |  |
| Secondary education/VWO | 1 (1.5%) | 19 (12.1%) |  |
| Higher vocational education/HBO | 6 (9.2%) | 57 (36.3%) |  |
| Income (*N* (%)) |  |  | *Χ^2^*(6)=112.110^***^ |
| Student grant | 1 (1.6%) | 21 (14.1%) |  |
| Social welfare provision | 18 (29.0%) | 0 (0%) |  |
| Unemployment benefit | 10 (16.1%) | 1 (0.7%) |  |
| Old age pension | 0 (0%) | 1 (0.7%) |  |
| Other benefit | 15 (24.2%) | 5 (3.4 %) |  |
| Income from paid employment | 17 (27.4%) | 121 (81.1%) |  |
| None | 1 (1.6%) | 0 (0%) |  |
| Ethnical background (*N* (%)) |  |  | *Χ^2^*(12)=18.824 |
| Dutch | 43 (66.2%) | 107 (68.2%) |  |
| Surinamese | 4 (6.2%) | 2 (1.3%) |  |
| Turkish | 1 (1.5%) | 0 (0%) |  |
| Moroccan | 0 (0%) | 1 (0.6%) |  |
| Dutch-Indian | 1 (1.5%) | 2 (1.3%) |  |
| Other | 2 (3.0%) | 7 (4.4%) |  |
| Missing | 14 (21.6%) | 38 (24.2%) |  |

*Note.* *SD*= standard deviation; *N*=number of participants; ^***^ *p*<.001.

**Table S2**

*Item Means, Standard Deviations and Factor Loadings of Grandiose and Vulnerable Narcissism of the Dutch Narcissism Scale*

| Grandiose Narcissism (*n*=217) | *M* | *SD* | Factor Loading |
| --- | --- | --- | --- |
| Compared to others, I’m not doing so badly in life. | 5.41 | 1.43 | .685 |
| Sometimes I think I'm happy with myself. | 4.60 | 1.68 | .638 |
| I see myself as someone with leadership abilities. | 5.05 | 1.69 | .594 |
| I feel that I regularly put people on the right track. | 4.94 | 1.28 | .592 |
| I can easily get others to do what I want. | 4.75 | 1.38 | .572 |
| I am usually well aware of the impression that people have of me. | 4.94 | 1.30 | .546 |
| Usually, I can have an idea of the motives of others. | 5.29 | 1.11 | .466 |
| I think it is important to reflect on myself to see how I experience all kinds of things exactly as I experience them. | 4.59 | 1.55 | .418 |
| I sometimes catch myself looking in window at myself. | 3.87 | 2.05 | .406 |
| I trust my own opinions and initiatives the most. | 5.18 | 1.32 | .390 |
| Others see me as someone who can stand up for themselves. | 5.81 | 1.16 | .374 |
| Sometimes I do something to see what effect it has on other people. | 3.97 | 1.81 | .224 |
| Vulnerable Narcissism (*n*=220) | *M* | *SD* | Factor Loading |
| I often take other people’s comments personally. | 3.55 | 1.79 | .732 |
| It's a bit disappointing that people don't always see who I really am. | 3.96 | 1.78 | .701 |
| I find it very annoying when people don't pay attention to who I really am. | 4.11 | 1.80 | .638 |
| Sometimes I get completely preoccupied with personal matters, health and my relationships with others. | 4.46 | 1.66 | .634 |
| When people really notice me, I get the feeling that I’m really alive. | 3.00 | 1.72 | .624 |
| Sometimes, small comments from others can easily hurt my feelings. | 4.02 | 1.80 | .614 |
| Sometimes I don't feel at ease until I know that there is at least one person who likes me. | 3.47 | 1.85 | .561 |
| When I enter a place, I am often painfully aware of the way others look at me. | 3.15 | 1.68 | .487 |
| It hurts when others don't treat you the way you think you should be treated. | 4.55 | 1.71 | .486 |
| I'd rather not admit it, but I do fish for compliments. | 3.83 | 1.77 | .452 |
| When I see myself in a photo, it's hard for me to get rid of that. | 2.68 | 1.62 | .444 |

*Note*. Extraction Method: Principal Component Analysis; *M*=Mean; *SD*=Standard Deviation; *n*=Number of valid cases.

**Table S3**

*Item Means, Standard Deviations and Factor Loadings of Self-Control and Identity Integration of the Severity Indices of Personality Problems – Short Form*

| Self-Control (*n*=217) | *M* | *SD* | Factor Loading |
| --- | --- | --- | --- |
| I have such strong feelings that I easily lose control of them.^*^ | 3.38 | .93 | .822 |
| I lose control sometimes to the extent that people are frightened of me.^*^ | 3.22 | 1.07 | .822 |
| I seem to do things that I regret more often than other people do.^*^ | 3.22 | .98 | .812 |
| I often act impulsively even though I know I will regret it later on.^*^ | 3.08 | 1.04 | .807 |
| I often cannot help expressing my moods inappropriately.^*^ | 3.26 | .97 | .793 |
| It is hard for me to control my aggression towards others.^*^ | 3.46 | .91 | .789 |
| I often overreact to minor problems.^*^ | 3.19 | .97 | .757 |
| The way I feel or behave is often very unpredictable.^*^ | 3.16 | 1.05 | .737 |
| Others have told me that I should try harder to avoid losing control over my feelings.^*^ | 3.41 | .92 | .732 |
| I frequently say things I regret later. ^*^ | 2.78 | .96 | .691 |
| Sometimes I get so overwhelmed that I can’t control my reactions.^*^ | 2.93 | 1.11 | .644 |
| I usually have adequate control over my feelings. | 3.31 | .88 | .582 |
| Identity Integration (*n*=218) | *M* | *SD* | Factor Loading |
| I often feel that my life is meaningless.^*^ | 3.53 | .87 | .803 |
| It is hard for me to really enjoy doing things.^*^ | 3.09 | 1.00 | .787 |
| I often feel that I am not as worthy as other people.^*^ | 3.46 | .92 | .772 |
| One of my problems is that I cannot easily let myself have a good time.^*^ | 3.31 | .98 | .766 |
| I am often confused about what kind of person I really am.^*^ | 3.22 | 1.05 | .751 |
| When I try to understand myself, I often get more confused than I was before.^*^ | 3.38 | .95 | .745 |
| I often see no reason to continue living.^*^ | 3.63 | .79 | .745 |
| Sometimes it seems that everything in me somehow blocks the capacity to have fun.^*^ | 3.43 | .92 | .736 |
| I strongly believe that life is worth living. | 3.50 | .89 | .701 |
| Overall I feel that my activities are enjoyable to me. | 3.43 | .85 | .652 |
| I usually have a low opinion of myself.^*^ | 3.19 | .99 | .636 |
| I strongly believe that I am just as worthy as other people. | 3.49 | .81 | .594 |

*Note*. Extraction Method: Principal Component Analysis; *M*=Mean; *SD*=Standard Deviation; *n*=Number of valid cases; ^*^Reverse-coded items.
